# Alzheimer’s Disease: Exploring Pathophysiological Hypotheses and the Role of Machine Learning in Drug Discovery

**DOI:** 10.3390/ijms26031004

**Published:** 2025-01-24

**Authors:** Jose Dominguez-Gortaire, Alejandra Ruiz, Ana Belen Porto-Pazos, Santiago Rodriguez-Yanez, Francisco Cedron

**Affiliations:** 1 Department of Computer Science and Information Technologies, Faculty of Computer Science, Universidade da Coruña, 15071 A Coruña, Spain; j.dgortaire@udc.es (J.D.-G.);; 2 Faculty of Biological Sciences, Universidad Central del Ecuador, Quito 170136, Ecuador; 3 Faculty of Odontology, UTE University, Quito 170902, Ecuador; 4 Faculty of Medical Sciences, Universidad Central del Ecuador, Quito 170136, Ecuador; 5 CITIC—Research Center of Information and Communication Technologies, Universidade da Coruña, 15008 A Coruña, Spain; 6 CITEEC—Center for Technological Innovation in Construction and Civil Engineering, Universidade da Coruña, 15008 A Coruña, Spain

**Keywords:** pathophysiology, neuroinflammation, therapeutic target, mitochondrial dysfunctions, machine learning, AI applications, virtual screening, molecular docking

## Abstract

Alzheimer’s disease (AD) is a major neurodegenerative dementia, with its complex pathophysiology challenging current treatments. Recent advancements have shifted the focus from the traditionally dominant amyloid hypothesis toward a multifactorial understanding of the disease. Emerging evidence suggests that while amyloid-beta (*A*β) accumulation is central to AD, it may not be the primary driver but rather part of a broader pathogenic process. Novel hypotheses have been proposed, including the role of tau protein abnormalities, mitochondrial dysfunction, and chronic neuroinflammation. Additionally, the gut–brain axis and epigenetic modifications have gained attention as potential contributors to AD progression. The limitations of existing therapies underscore the need for innovative strategies. This study explores the integration of machine learning (ML) in drug discovery to accelerate the identification of novel targets and drug candidates. ML offers the ability to navigate AD’s complexity, enabling rapid analysis of extensive datasets and optimizing clinical trial design. The synergy between these themes presents a promising future for more effective AD treatments.

## 1. Introduction

Alzheimer’s disease (AD) is a major progressive neurodegenerative disorder and common inborn form of dementia characterized by marked deficits in memory about other neurological features. Indeed, average global life expectancy has continued to rise in recent times [1]. The search for an effective treatment and therapeutic target is essential because there is no cure for AD, one of the major causes of senile dementia among about 35 million aged 60 years and above [2]. Recent progress in the pathophysiology of AD and its relationship with the use of artificial intelligence (AI) algorithms in drug discovery has resulted in a promising number of new AD therapeutic targets and strategies [3].

Alzheimer’s disease is an illness that involves a long period of asymptomatic progression that leads to a progressive, perhaps decade-long duration, culminating in cognitive, social, and occupational impairment, finally resulting in death [4]. Regarding the basic molecular mechanisms of Alzheimer’s, it is characterized by the accumulation of abnormal proteins in the brain that impair its function [5]. The two main proteins involved, *A*β and tau, act as toxins inside nerve cells, disrupting communication between neurons and eventually causing neuronal death [6]. This leads to symptoms of cognitive decline, such as memory loss and confusion.

Traditional AD treatments target reducing symptoms but do not stop the progression of the disease [7]; among the most accepted of these is the inhibition of acetylcholinesterase and butyrylcholinesterase, due to the degradation of acetylcholine, which is a neurotransmitter whose levels decrease in Alzheimer’s patients. This inhibition causes an increase in the availability of this compound, which improves the cognitive capacity of the patients [8]. Some of the drugs that are used are donepezil, rivastigmine, and galantamine, although their efficiency is limited and they have low specificity [9].

The drug discovery for AD has had a failure rate of as high as 99.6% in clinical trials of new medications [10]. The newest class of approved drug is aducanumab and lecanemab, which are monoclonal antibodies targeting Aβ solubility [11]. These drugs are in phase III clinical trial on early-stage patients, which found that, regarding the progression of brain amyloid deposits accumulating in patients, those that received aducanumab accumulated significantly lower amounts than those that received the placebo. However, after the approval of the medication, some controversy was made because a few experts had doubts about whether the clinical trial was valid and whether the medicine was efficacious in real clinics [12]. The bibliography suggests that the accumulation of Aβ is not the event causing the disease but rather a compensatory process [13]. The initial step in the development of the disease is a gap in the knowledge of the psychopathology of Alzheimer’s disease; there are several hypotheses about its onset [14]. Many hypotheses have been postulated in recent years. Some of them are the amyloid hypothesis, the cholinergic hypothesis, the tau hypothesis, other neuroinflammation hypotheses, the mitochondrial dysfunction hypothesis, environmental risks, and genetics. This implies that the origin of the disease is multicentric and is not yet defined [15].

Some of the most recent developments in the pathophysiology of AD are explained by the possible employment of new technological advancements, including AI, which now bring with them exciting new opportunities in developing new therapeutic targets and strategies [16]. There is, therefore, a growing interest in the targeting of Aβ plaques acting upstream of tau pathology, very early in AD onset, i.e., before massive damage of the neurons. It has since led to the identification of new potential therapeutic targets, such as enzymes responsible for the production of Aβ and tau proteins, and new approaches to modulate inflammation and mitochondrial dysfunction [17].

AI was also applied in the area of drug development and demonstrated great potential to speed up the process of the invention and development of new treatments for AD [18]. Meanwhile, AI-assisted clinical trials make it possible to reveal the most effective population for new therapies among the patient population. AI-based drug discovery allows for the screening of huge numbers of compounds with a high amount of efficiency. Indeed, in individualized medicine, AI indicates a consequence for each patient by individualizing treatments using personal patient characteristics [19].

Since the impact of AD is very remarkable on the patients, their families, and, in general, on society, the most emphatic discussion in this review is how to develop effective therapies for this devastating disease [20]. It has not only identified new therapeutic targets but also now holds new promise for the development of effective treatments for AD, using AI in drug development [21]. This scientific review article is hence a summary of the most recent research work on the hypothesis of AD development and new drugs for the treatment of this devastating disease.

## 2. Hypothesis of the Development of Alzheimer’s Disease

Alzheimer’s disease stands as one of the most intricate and distressing forms of neurodegenerative disorders, marking its presence as the leading cause of dementia globally, impacting millions [22]. The disease’s pathophysiology is notably complex and multifaceted, underscored by the accumulation of various abnormal proteins within the brain.

Although its etiology is not fully understood yet, extensive research has identified several hypotheses that attempt to explain the underlying mechanisms of the disease. The most studied hypotheses include the amyloid hypothesis, the tau hypothesis, the neuroinflammation hypothesis, and the mitochondrial dysfunction hypothesis, among others [23].

Recent studies have further suggested the involvement of the gut–brain axis in Alzheimer’s disease progression and development, with research indicating that alterations in gut microbiota composition can influence brain function and cognitive performance [24].

### 2.1. The Amyloid Hypothesis

The amyloid hypothesis posits that the aggregation of Aβ peptides in the brain leads to the formation of senile plaques, subsequently triggering neuroinflammation, oxidative stress, and synaptic dysfunction. This cascade eventually results in neuronal death and the cognitive decline characteristic of Alzheimer’s disease, although some recent studies have questioned this hypothesis, suggesting that Aβ accumulation might be a consequence of other pathological processes rather than being the primary cause [25].

Emerging evidence has unveiled different forms of Aβ, implicating it in novel therapeutic targets. For instance, it is now believed that small soluble oligomers of Aβ, rather than plaques, are the major toxic species in Alzheimer’s disease. Moreover, new enzymes and pathways involved in Aβ’s production and clearance have been identified, opening new avenues for drug development [26].

Despite being a central theory for decades, the role of the amyloid hypothesis in Alzheimer’s disease pathology continues to evolve, revealing complex dynamics and suggesting new potential therapeutic targets, underscored by the promising role of AI in drug development [27].

### 2.2. Tau Hypothesis

The tau hypothesis suggests that the accumulation of abnormal tau protein forms in the brain is one of the principal causes of Alzheimer’s disease. Tau, a vital protein for nerve cell structure and function, stabilizes microtubules in healthy neurons—structures that act as cellular highways for nutrient and molecule transport [28].

In Alzheimer’s, the tau protein becomes abnormal and forms clumps called neurofibrillary tangles, disrupting microtubules and leading to nerve cell death, contributing to cognitive impairment [29].

Recent evidence has strengthened the tau hypothesis, identifying specific abnormal tau forms linked to disease progression. Research is ongoing for treatments targeting abnormal tau to prevent its accumulation in the brain [30]. Advances in imaging techniques now allow for tau visualization in living patients, promising new diagnostic and treatment tools for Alzheimer’s disease [31].

The tau hypothesis remains a promising avenue for the development of new treatments for Alzheimer’s disease. However, further research is needed to fully understand the role of the tau protein in the disease and to identify effective therapeutic targets for its treatment.

### 2.3. Neuroinflammation Hypothesis

This hypothesis highlights inflammation’s critical role in Alzheimer’s disease pathogenesis, proposing that chronic inflammation drives the disease’s development and progression. Elevated inflammatory mediators, such as cytokines and chemokines, in Alzheimer’s patient’s brains may damage neurons and impair cognition [32].

Microglia, the resident immune cells of the CNS, play a dual role in AD pathophysiology. Under normal conditions, they actively survey the CNS environment, clearing debris and misfolded proteins. In early AD stages, they can even help to reduce Aβ and tau pathology. Recent studies suggest that microglia have a potential protective role in reducing Aβ plaques by phagocytosing these aggregates, thus conferring a neuroprotective effect [33].

However, as AD progresses, sustained exposure to elevated Aβ levels, hyperphosphorylated tau, and other damage-associated molecular patterns (DAMPs) can induce microglial overactivation [34]. Overactivated microglia release large quantities of pro-inflammatory cytokines, chemokines, and reactive oxygen species, further aggravating neuronal injury and amplifying neurodegenerative cascades [35].

Moreover, microglial reactivity in advanced AD has been associated with a dysfunctional phenotype, characterized by the inefficient clearance of Aβ and increased secretion of neurotoxic mediators [33]. This shift from a protective to a detrimental underscores the complexity of microglial function in AD and highlights the importance of timing and context in therapeutic interventions aimed at modulating microglia [36].

In addition, there is growing interest in the role of the gut microbiome in the pathogenesis of AD and its potential as a therapeutic target. Alterations in the gut microbiome can lead to inflammation and immune dysregulation, which may contribute to the pathogenesis of AD. Modulation of the gut microbiome through diet or the use of probiotics may have prospects in the prevention and treatment of AD [37].

Novel therapeutic targets aimed at modulating the immune response in AD include targeting the receptor for advanced glycation end products and specific cytokines or chemokines that activate microglia [38].

### 2.4. Mitochondrial Dysfunction Hypothesis

Mitochondria are essential organelles that play a crucial role in cellular metabolism and energy production [39]. There is increasing evidence that mitochondrial dysfunction is a critical factor in AD pathogenesis. Studies have reported several mitochondrial abnormalities in cell brains, which include a reduction in mass, alterations in morphology, and impairment in mitochondrial respiration [40]. All of these have been linked to oxidative stress, inflammation, apoptosis, and other cellular processes that contribute to the neurodegeneration in AD [41].

Compounding these effects, oxidative stress, mitochondrial dysfunction, and copper dysregulation are tightly interwoven in AD. Elevated copper levels worsen mitochondrial damage by disrupting electron transport, increasing reactive oxygen species (ROS), and impairing energy metabolism. This synergy not only amplifies oxidative stress but also promotes the aggregation of both Aβ and tau proteins, thereby hastening neurodegeneration [42]. Therefore, mitigating copper toxicity and its downstream impacts on mitochondria holds promise as a therapeutic avenue for slowing AD progression.

Recent studies have pointed to several potential therapeutic targets for AD based on the hypothesis of mitochondrial dysfunction. These include modulators of mitochondrial function, e.g., antioxidants, inducers of mitochondrial biogenesis, and enhancers of mitophagy. For instance, recent findings have shown that the induction of mitochondrial biogenesis via the PGC-α pathway improved mitochondrial function and diminished Aβ pathology in murine models of AD [43]; likewise, antioxidants such as MitoQ and SS-31 have been reported to protect against mitochondrial dysfunction and to diminish cognitive decline in animal models of AD [44].

Other novel therapeutic targets include modulators of mitochondrial dynamics and mitophagy, such as Mdivi-1 and Urolithin A, respectively. Indeed, Mdivi-1, a small molecule inhibitor of Drp1, can regulate mitochondrial fission, reportedly improving mitochondrial function and reducing beta-amyloid pathology in mouse models of AD [45] and Urolithin A, a natural compound that has been reported to induce mitophagy and to improve mitochondrial function in AD models [46].

Understanding the evidence behind and against the role of mitochondrial dysfunction in AD as well as the development of safe and effective therapies targeting these pathways will be necessary to further this approach to the discovery of novel AD therapeutics.

### 2.5. Other Hypotheses for the Development of Alzheimer’s

In the field of Alzheimer’s disease research, several hypotheses have been proposed to account for the complex nature of Alzheimer’s disease in this field over the years: the amyloid, the tau, the neuroinflammation, and the mitochondrial dysfunction hypotheses, as well as the atherosclerotic plaques hypothesis, to name a few. Others have been proposed recently to provide further insight into the underlying mechanisms driving the onset and progression of Alzheimer’s disease [47].

One of these is the synaptic dysfunction hypothesis, which postulates that alterations in synaptic function and communication between neurons play a crucial role in the development and progression of AD [48]. Several studies have highlighted the involvement of synapses in the pathogenesis of AD, suggesting that restoring synaptic function may represent a possible therapeutic target [49].

Another hypothesis is the oxidative stress theory, which suggests that increased levels of reactive oxygen species (ROS) and the resulting oxidative damage play a critical role in the development and progression of the disease [50]. ROS are highly reactive molecules that can damage essential cellular components such as lipids, proteins, and DNA, leading to impaired neuronal function and cell death [51]. This cellular state is related to other pathological processes, such as amyloid-beta aggregation and tau hyperphosphorylation, further exacerbating neuronal dysfunction [52,53].

The neurovascular hypothesis claims that cerebral blood flow and vascular modifications play a role in the onset and progression of Alzheimer’s disease. This has been corroborated by accumulating evidence demonstrating, for example, blood–brain barrier dysfunction, which reduces cerebral blood flow and impairs vascular function in in Alzheimer’s disease. Clinical studies have also shown that cerebral blood flow takes place in Alzheimer’s disease [54].

The endoplasmic reticulum stress hypothesis concerns protein synthesis and folding. This hypothesis posits that endoplasmic reticulum stress, brought on by the accumulation of misfolded proteins, may cause the demise of neurons in Alzheimer’s disease [55].

There is also the hypothesis of epigenetics, which refers to changes in gene expression that are not related to changes in the DNA sequence. This hypothesis suggests that epigenetic modifications, such as DNA methylation and histone modification, may contribute to the development of Alzheimer’s by altering the expression of genes that are important for neuronal function [56].

Autophagy is a cellular process that eliminates damaged proteins and dysfunctional organelles [57]. This hypothesis suggests that autophagy dysfunction may contribute to the accumulation of abnormal proteins and neuronal death in Alzheimer’s disease [58].

Other hypotheses are the zinc and aluminum hypotheses. Zinc is an essential metal that plays an important role in neuronal function. At dysfunction, the metabolism of zinc may contribute to the development of Alzheimer’s disease by increasing the production of amyloid-beta and tau [59]. The same is argued for the exposure to aluminium, which may increase the production of amyloid-beta and tau [60].

Lifestyle factors, such as diet, activity, and sleep, influence the risk of Alzheimer’s [61].

In summary, these emerging hypotheses add detail to the multifaceted nature of Alzheimer’s disease and provide potentially novel therapeutic targets for drug development. Additional research is required to fully understand the mechanisms underlying these hypotheses and their potential implications for Alzheimer’s treatment.

### 2.6. Unification Hypothesis

**Figure 1:**
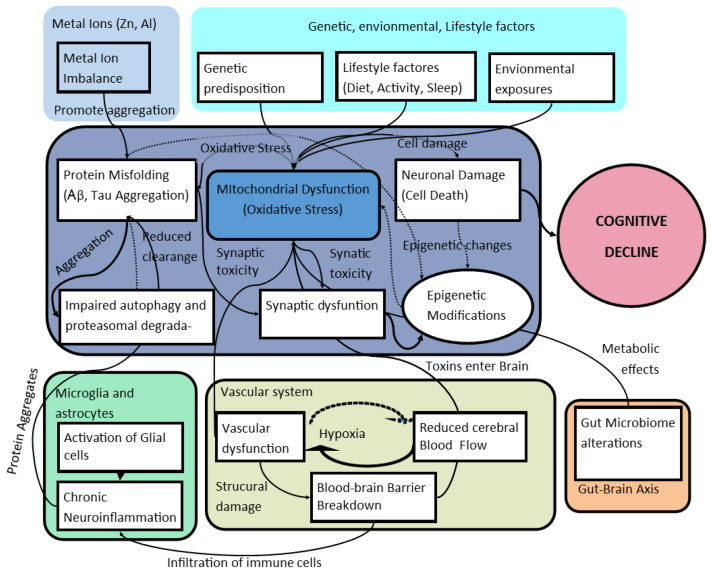
In this figure, the bold lines illustrate direct relationships or causal connections between the various pathogenic factors in Alzheimer’s disease, while the dotted lines denote feedback loops and amplification cycles that further exacerbate existing pathological processes. Overall, this schematic integrates multiple interdependent mechanisms, highlighting how genetic, environmental, and lifestyle factors converge to initiate mitochondrial dysfunction and oxidative stress, ultimately promoting amyloid-beta and tau protein aggregation. These misfolded proteins trigger synaptic dysfunction and neuronal death while also activating microglia and astrocytes that mount an inflammatory response, which, if sustained, amplifies protein aggregation and accelerates neurodegeneration. Moreover, vascular abnormalities and gut microbiome alterations potentiate both inflammation and oxidative damage, and imbalances in metal ions further promote pathological protein aggregation. Impaired autophagy and epigenetic modifications compound disease progression by hindering the clearance of toxic species and altering gene expression, respectively. By illustrating these interconnected pathways and feedback loops, the figure underscores the multifactorial nature of Alzheimer’s disease and the potential benefit of targeting multiple nodes in this network for therapeutic intervention.

The complexity of Alzheimer’s disease has led to the proposal of a unification hypothesis that seeks to integrate various perspectives on the disease’s pathogenesis. This hypothesis aims to explain the interactions between amyloid-β accumulation, tau protein pathology, neuroinflammation, mitochondrial dysfunction, and other relevant factors, offering a comprehensive understanding of AD (Figure 1). Recent research suggests that these mechanisms are not mutually exclusive but are instead interconnected processes contributing to the disease’s onset and progression [62].

Aβ accumulation has long been considered a hallmark of AD, with its aggregation leading to plaque formation and subsequent neuronal damage. However, recent studies indicate that Aβ may also interact with tau protein and intensify tau pathology [63]. Tau protein, normally involved in stabilizing microtubules, becomes hyperphosphorylated and forms neurofibrillary tangles in AD, disrupting cellular transport mechanisms and leading to neuronal death [64]. The interplay between Aβ and tau is thought to amplify neurodegenerative processes, creating a vicious cycle of neuronal damage.

Neuroinflammation is another critical component of the unification hypothesis. Chronic inflammation in the brain, driven by the activation of microglia and astrocytes, contributes to AD progression [6]. These glial cells, in response to Aβ and tau pathology, release pro-inflammatory cytokines and reactive oxygen species, further damaging neurons and promoting a toxic environment [65]. Additionally, recent studies have highlighted the role of the gut microbiome in modulating neuroinflammatory responses, suggesting that systemic inflammation might also influence AD pathogenesis.

Mitochondrial dysfunction is increasingly recognized as a significant factor in AD. Mitochondria, the powerhouses of the cell, are responsible for energy production and regulating apoptosis. In AD, mitochondrial abnormalities, including impaired respiratory function and increased oxidative stress, have been observed [66]. These dysfunctions not only contribute to neuronal energy deficits but also enhance the production of reactive oxygen species, which can damage cellular components and promote neuroinflammation and protein aggregation.

Emerging evidence also points to the role of vascular contributions in AD. Vascular dysfunction, including reduced cerebral blood flow and blood–brain barrier breakdown, may lead to hypoxia and the accumulation of neurotoxic substances in the brain [67]. This vascular impairment can enhance Aβ deposition and tau pathology, further intertwining with neuroinflammatory and mitochondrial pathways.

While multiple hypotheses have attempted to explain the complex pathophysiology of AD, there remains a lack of conceptual integration that explains how these processes interact in a single narrative. We propose a new integration hypothesis based on the synchronization of neuroinflammatory events with mitochondrial dysfunction, which could trigger the synaptic collapse observed in early stages of the disease [68]. Rather than viewing these pathological routes independently, we suggest that chronic inflammation activates dysfunctional mitochondria, causing a positive feedback loop of oxidative stress that amplifies tau and Aβ toxicity. This hypothesis posits that therapeutic interventions designed to regulate the dialogue between microglia and mitochondria could offer a promising pathway to slow the progression of neurodegeneration.

This unification hypothesis underscores the importance of a holistic approach to understanding AD. By considering the interdependence of these pathological mechanisms, researchers can develop more effective therapeutic strategies. For instance, targeting multiple pathways simultaneously—such as mitigating neuroinflammation, improving mitochondrial function, and reducing Aβ and tau pathology—could provide synergistic effects in slowing or halting disease progression [67]. Additionally, novel therapeutic approaches that address vascular health and the gut–brain axis might offer new avenues for intervention.

Table 1 provides a comprehensive summary of the various hypotheses proposed for the development of Alzheimer’s disease, highlighting their key mechanisms, supporting evidence, and potential therapeutic targets. This consolidation underscores the multifactorial nature of AD, suggesting that multiple interconnected pathways contribute to its onset and progression. Recognizing the interplay between these mechanisms, as emphasized by the unification hypothesis, is crucial for developing holistic and effective therapeutic strategies. By targeting multiple pathways simultaneously—such as reducing amyloid and tau pathology, mitigating neuroinflammation, and enhancing mitochondrial function—there is potential to create synergistic treatments that more effectively slow or halt disease progression. This integrated approach holds promise for improving clinical outcomes and offers a more profound understanding of Alzheimer’s disease pathogenesis.

#### Pathway of Unification Hypothesis

The proposed pathway of the unified hypothesis, illustrated in Figure 1, begins with a combination of genetic predispositions, environmental exposures, and lifestyle factors such as diet, physical activity, and sleep patterns [61]. These factors can lead to initial metabolic disturbances, including mitochondrial dysfunction and oxidative stress [39]. Mitochondrial dysfunction results in impaired energy production and increased production of reactive oxygen species, exacerbating oxidative stress [66].

Oxidative stress contributes to the misfolding and aggregation of Aβ peptides and hyperphosphorylated tau proteins [52]. The accumulation of these abnormal proteins leads to the formation of amyloid plaques and neurofibrillary tangles, key pathological features of AD [64].

The presence of Aβ and tau aggregates activates microglia and astrocytes, the brain’s resident immune cells, triggering a chronic neuroinflammatory response [32]. Neuroinflammation further promotes neuronal damage and synaptic dysfunction, disrupting communication between neurons [65].

Vascular dysfunction, including reduced cerebral blood flow and blood–brain barrier breakdown, may contribute to hypoxia and facilitate the entry of peripheral inflammatory mediators and toxins into the brain, exacerbating neuroinflammation and protein aggregation [54].

Impaired autophagy and proteasomal degradation systems hinder the clearance of misfolded proteins and damaged organelles, allowing toxic aggregates to accumulate [58]. Epigenetic modifications may alter gene expression patterns involved in neuronal survival, synaptic function, and protein homeostasis, further promoting disease progression [56].

Metal ions such as zinc and aluminum may influence amyloid-beta and tau pathology by affecting protein aggregation and toxicity [59,60]. Additionally, alterations in the gut microbiome can modulate systemic inflammation and impact brain function via the gut–brain axis [24].

These interconnected processes create a vicious cycle of neuronal damage, synaptic loss, and cognitive decline characteristic of Alzheimer’s disease. By understanding this complex pathway, researchers can target multiple aspects of the disease simultaneously, potentially developing more effective therapeutic strategies.

## 3. Emerging Therapeutic Targets in Alzheimer’s Disease Research

Recent years have seen an explosion of research into the underlying mechanisms of Alzheimer’s disease, leading to new insights and potential therapeutic targets. Studies on amyloid and tau proteins have revealed new approaches for disease-modifying therapies, while neuroinflammation and mitochondrial dysfunction have emerged as promising areas of research [71]. Recent studies on amyloid and tau proteins have provided new insights into AD pathogenesis and potential therapeutic targets. Aβ and tau are the main components of amyloid plaques and neurofibrillary tangles, respectively, which are the hallmark pathological features of AD. Recent research has focused on identifying new ways to reduce the accumulation of Aβ and tau in the brain [72] One strategy is to target the enzymes responsible for the production of Aβ and tau. Beta-secretase and gamma-secretase are enzymes involved in Aβ formation and are promising targets for drug development. Several inhibitors have been developed and are being tested in clinical trials [11]. Similarly, the inhibition of tau kinases, such as GSK-3β, has shown promise in reducing tau pathology in animal models of AD [73]. Another approach is to enhance the clearance of Aβ and tau from the brain. One way to achieve this is to act on the immune system. Microglia, the brain’s immune cells, can engulf and eliminate Aβ and tau; however, in AD, microglia become dysfunctional and are unable to effectively eliminate these proteins. Recent research has focused on the development of drugs that can modulate the activity of microglia to improve their ability to clear Aβ and tau [74]. In addition, there is growing evidence that Aβ and tau may not act alone in the pathogenesis of AD and that other factors may be involved. Overall, recent studies on amyloid and tau proteins have led to the identification of new therapeutic targets for AD, including enzymes involved in the production of Aβ and tau [20].

One area of interest has been the role of inflammation in Alzheimer’s disease. Inflammation is a natural response of the immune system to injury or infection, but chronic neuroinflammation may contribute to brain neurodegeneration. Recent studies have shown that neuroinflammation is a key feature of Alzheimer’s disease [75]. Recent studies have shown that certain immune cells, such as microglia and astrocytes, play a key role in regulating the inflammatory response in the brain. Dysregulation of these cells may contribute to neuroinflammation and the subsequent development of Alzheimer’s disease [76]. In recent years, new discoveries have emerged about the role of inflammation in Alzheimer’s disease. Inflammation has been identified as a key factor in the development and progression of Alzheimer’s disease, and it is now understood that chronic inflammation can lead to the accumulation of Aβ plaques and tau tangles in the brain [77]. New evidence suggests that targeting neuroinflammation may be a viable therapeutic strategy for Alzheimer’s disease. Several anti-inflammatory drugs, such as non-steroidal anti-inflammatory drugs (NSAIDs) and minocycline, have been investigated for their potential to slow or prevent disease progression; however, these drugs have been limited by their side-effect profile and lack of specificity for brain inflammation [78].

More recent research has also focused on identifying specific targets within the inflammatory pathway that can be selectively targeted. For example, the complement system is involved in neuroinflammation and has been the subject of preclinical studies, with promising results [79]. Other targets include cytokines and chemokines, small signalling molecules involved in inflammation. In addition, research has identified specific inflammatory pathways, such as the NLRP3 inflammasome, as potential targets for intervention [80].

In addition to hypotheses related to amyloid, tau, and inflammation, new research has implicated mitochondrial dysfunction in Alzheimer’s disease [81]. Mitochondria are organelles responsible for energy production within cells and are essential for cell function. Dysfunctional mitochondria can cause cell damage and death, and recent evidence suggests that mitochondrial dysfunction may play a role in the pathogenesis of Alzheimer’s disease [82].

Studies have shown that mitochondrial dysfunction is present both in the brains of Alzheimer’s patients and in animal models of the disease. Dysfunctional mitochondria in AD have been shown to produce less energy, generate more ROS, and have impaired calcium homeostasis, leading to neuronal death. In addition, studies have suggested that amyloid and tau pathologies may directly contribute to mitochondrial dysfunction in Alzheimer’s disease [83].

In light of these findings, treatment of mitochondrial dysfunction has emerged as a possible therapeutic strategy for Alzheimer’s disease. Several studies have explored the use of mitochondria-targeted antioxidants, such as MitoQ, as a potential therapy for Alzheimer’s disease. Other studies have investigated the potential of compounds that may improve mitochondrial function, such as the AMPK activator metformin [84].

One of the emerging areas of research is the role of the gut microbiome in Alzheimer’s disease, as recent studies have shown that changes in the gut microbiota can lead to alterations in the brain associated with cognitive impairment and dementia. Other research has focused on the role of the microbiome as a modulator of the immune system in Alzheimer’s disease, in particular, the activation of microglia, and the potential therapeutic benefits of modulating the immune response in the brain [85].

Other emerging areas of research include the role of epigenetic factors in Alzheimer’s disease, as well as the potential benefits of lifestyle interventions, such as exercise and dietary modifications, in reducing the risk of developing the disease [70]. These findings offer new insights into the mechanisms underlying the development of Alzheimer’s disease and may lead to the identification of new therapeutic targets for the treatment and prevention of the disease.

Current theories about Alzheimer’s offer a fragmented view of its etiology. By integrating recent evidence, we propose that future research should focus on the link between neuroinflammation and mitochondrial alterations as the primary drivers of the disease. Studies in animal models suggest that regulating inflammatory activity could improve mitochondrial function and thus preserve neuronal synapses [69]. This approach implies a promising direction in the search for new therapeutic targets that act on multiple pathological processes simultaneously, a task in which AI could facilitate the analysis of large volumes of clinical and experimental data.

## 4. The Use of Artificial Intelligence in Alzheimer’s Research

The complexity of neurodegenerative diseases such as Alzheimer’s disease presents significant challenges in biomedical research. Traditional approaches often fail to handle the sheer volume of data generated from genetic, proteomic, and clinical studies. AI has emerged as a powerful tool to address these challenges. Its capacity to process large amounts of data, identify patterns, and simulate biological processes is transforming the way that biomedical research is carried out [86]. The use of ML algorithms and deep learning networks is particularly crucial in improving early diagnosis, identifying novel therapeutic targets, and enhancing the efficiency of drug development.

Some of the examples of success in the early diagnosis of Alzheimer’s disease demonstrate how ML models leverage multimodal data to enhance diagnostic accuracy and offer insights into disease progression mechanisms.

One of them is a novel deep learning framework integrating 3D vision transformers (3D-ViTs) and deep belief networks (DBNs) for the early detection of Alzheimer’s disease. The model analyzes structural MRI data divided into 138 brain regions (regions of interest, ROIs) from the ADNI dataset (532 samples: 168 cognitively normal, 247 mild cognitive impairment, and 117 Alzheimer’s disease). It achieves high accuracy (up to 90% for AD vs. CN classification) and interpretability, highlighting brain regions contributing significantly to disease progression. The approach demonstrates robustness and scalability, emphasizing its utility in clinical applications [87].

Another study has developed a multimodal machine learning model (EDAMM) combining acoustic and linguistic features for Alzheimer’s disease diagnosis. Using datasets ADReSSo (166 samples) and NCMMSC2021 (long speech: 280 training, 119 test samples), the model applies Wav2Vec2.0, TF-IDF, and Word2Vec for feature extraction. Self-attention and cross-modal attention mechanisms enhance feature integration, achieving accuracies of 86.7% (ADReSSo) and 90.2% (NCMMSC2021). The study highlights the effectiveness of combining speech and text data for non-invasive, cost-effective early AD diagnosis [88].

AI has revolutionized the study of neurodegenerative diseases through the use of various techniques, including different types of learning: supervised, unsupervised, and reinforcement learning, as described in Table 2. The most commonly used techniques are deep learning models that use the machine learning models described above; all AI approaches are summarized in Table 3. In biomedical research, the impact of AI has been profound, enabling better identification of disease biomarkers and new therapeutic compounds. However, challenges such as data heterogeneity and a lack of labeled data in the early stages of Alzheimer’s pose obstacles to the broader implementation of AI in this pathology [89]. However, integrating AI with precision medicine holds great potential for future therapeutic advances.

### 4.1. Virtual Screening of Active Compounds Targeting Therapeutic Sites

Virtual screening is a computational method used to search large chemical libraries to identify potential bioactive compounds that could act as drugs or potential drugs [105]. The main goal is to reduce the number of molecules that need to be tested experimentally, saving time and resources.

Ligand-based virtual screening (LBVS): This method uses information from known active ligands to find compounds with similar structures or chemical properties [106]. It assumes that molecules with similar structures will exhibit similar biological activity [107]. LBVS is particularly effective when the 3D structure of the target is unknown, and relies on chemical fingerprints or molecular descriptors to find potential compounds.

Machine learning algorithms, such as support vector machines (SVMs), random forests (RFs), artificial neural networks (ANNs), and deep neural networks (DNNs), are commonly used to identify potentially active compounds [108]. These tools accelerate the identification of key molecular features that correlate with biological activity, making them a rapid and highly effective avenue for potential Alzheimer’s drug discovery.

AI can be used to facilitate the identification of new therapeutic targets for Alzheimer’s. By mining data from genomic and proteomic datasets, AI tools such as DeepBind can predict protein–DNA interactions, helping to identify genes involved in Alzheimer’s pathology [109]. Furthermore, AI models integrate vast molecular databases with advanced neural networks, such as graph convolutional networks, to predict molecular interactions [110]. These tools improve the virtual screening process by evaluating molecular properties and interactions more comprehensively.

After the virtual screening process, the efficacy of the selected compounds must be evaluated. First, it is important to evaluate the interaction of the ligands with the therapeutic targets through molecular docking and molecular dynamics [111]. After that, both in vitro and in vivo evaluations are performed. AI helps to optimize high-throughput screening and preclinical testing by predicting the outcomes of these assessments, streamlining the transition from virtual models to laboratory testing [112].

### 4.2. Advantages of AI-Assisted Molecular Docking and Molecular Dynamics

Molecular docking is a key technique in Alzheimer’s drug discovery, allowing us to predict how ligands interact with biological targets such as acetylcholinesterase or amyloid-beta plaques. AI has improved this process by optimizing ligand–receptor interaction prediction and improving the accuracy of docking simulations [113].

Molecular dynamics simulations, which predict the behavior of molecular complexes over time, have also benefited from advances in AI. Deep learning tools such as AlphaFold enable 3D models of proteins that are not described experimentally, leading to more accurate simulations of protein–ligand interactions by more effectively predicting protein structures [114].

These AI-powered simulations provide deeper insights into the dynamics of molecular complexes, critical to understanding drug efficacy in Alzheimer’s. Traditional docking and simulation methods often fail to accurately model the flexibility of biological systems. AI-powered techniques overcome these limitations by dynamically predicting changes in protein–ligand interactions and providing more realistic simulations [115].

Notably, models such as DeepDock and AtomNet use deep learning frameworks to improve the docking process. DeepDock improves the prediction of binding poses by learning from large datasets of protein–ligand complexes, while AtomNet applies CNNs to predict molecular binding affinities, considering the spatial arrangement of atoms [116,117].

Other models such as CNNScore and RF-Score integrate machine learning with traditional scoring functions to refine docking accuracy [118]. These models represent significant advances in leveraging AI to obtain more reliable molecular docking results.

Following AI-based predictions, preclinical studies validate the findings, ensuring that in silico models align with in vivo biological behavior.

### 4.3. Modeling the Interaction of Key Hypotheses in Alzheimer’s Development Using AI

Alzheimer’s disease is driven by several inter-related hypotheses, including amyloid-beta accumulation, tau hyperphosphorylation, and neuroinflammation [119] as the unifying hypothesis of Alzheimer’s disease development and the synchronization of neuroinflammatory events with mitochondrial dysfunction.

AI makes it possible to model these complex processes, integrating them into a multi-hypothesis framework to predict disease progression and identify new therapeutic targets. Through in silico simulations, AI tools allow researchers to explore the interactions between these hypotheses, offering insights into disease mechanisms that would be difficult to achieve through experimental studies alone [120].

Several AI-driven models have been developed to simulate these biological interactions. Systems biology models such as EpiSim integrate various molecular and cellular pathways to model disease dynamics [121]. Agent-based models such as Repast Simphony simulate single entities (e.g., cells or proteins) to study their interactions over time, allowing for the exploration of processes such as amyloid-beta aggregation [122]. Furthermore, Bayesian networks have been applied to Alzheimer’s disease to infer probabilistic relationships between different hypotheses, such as the interaction between tau protein pathology and amyloid-beta accumulation [123].

In addition to these modeling approaches, AI is being integrated into clinical trials to deliver personalized treatments tailored to individual patient profiles. For example, the trial “Design of Personalized Supplements Based on the Gut-Brain Axis to Reduce Alzheimer’s Disease Risk” (NCT06199193) leverages AI to optimize dietary interventions based on gut microbiota analysis [124]. Similarly, the “BRAIN App” trial (NCT06298474) uses AI-powered behavioral interventions to provide customized cognitive stimulation, aiming to enhance patient outcomes through individualized care [125]. These studies underscore the transformative potential of AI in combining biological modeling with personalized therapeutic strategies.

For example, reinforcement learning has been employed to model oxidative stress feedback loops, providing a deeper understanding of how oxidative damage exacerbates tau protein pathology [126]. DeepRL has shown potential to optimize treatment strategies by simulating how therapeutic interventions affect this positive feedback loop, as we propose in the unification hypothesis [127].

AI-generated models should be validated using experimental data, ensuring that they reflect the current biological processes underlying Alzheimer’s progression [128]. Once validated, AI models can simulate the effects of various therapeutic interventions, offering a pathway to optimize treatment strategies based on real-world data [129].

A promising approach to understanding the complex interactions between multiple Alzheimer’s hypotheses, such as neuroinflammation, mitochondrial dysfunction, and oxidative stress, would be to develop a multi-scale systems biology model that integrates these processes into a cohesive framework [130]. This could be achieved using a tool such as COPASI (Complex Pathway Simulator) version 4.39 (available at https://copasi.org/), which allows for modeling biochemical pathways and feedback loops [131], including the interaction between oxidative stress and mitochondrial dysfunction.

By incorporating AI algorithms such as dynamic Bayesian networks and deep reinforcement learning, this model could dynamically simulate how these processes influence each other over time [132]. Specifically, it would allow for observing positive feedback loops between neuroinflammation, mitochondrial dysfunction, and oxidative stress, helping to identify key intervention points to disrupt these cycles.

### 4.4. AI Applications in Patient Monitoring in Clinical Studies

Artificial intelligence technologies have transformed medicine and the way that data are collected and analyzed in Alzheimer’s clinical trials. Natural language processing (NLP), sensor-based monitoring, and wearable devices enable real-time data collection, which AI algorithms can analyze to track disease progression in a more accurate and individualized way [133]. For example, the digital platform Altoida uses a combination of smartphone-based cognitive assessments and AI algorithms to predict the onset of Alzheimer’s by analyzing daily behaviors and cognitive tasks [134]. Similarly, the Neurotrack app uses eye-tracking technology combined with AI to assess memory and cognitive function remotely, allowing researchers to collect real-time data on cognitive decline.

By continuously monitoring cognitive function, behavior, and daily activities, AI-powered tools generate longitudinal datasets that provide deeper insights into a patient’s condition over time [135]. This real-time data analysis can be used to tailor treatment plans based on a patient’s unique needs, improving the overall effectiveness of interventions and personalized medicine. The RADAR-AD (Remote Assessment of Disease And Relapse–Alzheimer’s Disease) project is another example of using wearables, mobile technology, and AI to track disease progression in real-world settings [136]. RADAR-AD collects data on movement, sleep, and other daily activities to provide insights into how Alzheimer’s affects daily life and tailor treatments accordingly [137].

AI’s predictive capabilities are also crucial in clinical trials, allowing researchers to forecast patient responses to treatments. Predictive models, such as survival analysis and time series algorithms, help to identify which patients will benefit most from experimental therapies. For example, IBM Watson for Clinical Trials uses AI to analyze clinical trial data and predict which patients are most likely to respond to specific treatments based on their genetic and clinical profiles [138]. AI tools also help tp monitor treatment adherence, providing a holistic view of how patients respond to therapy and how these responses impact their quality of life [139]. The AiCure platform, which uses computer vision and AI to monitor medication adherence using smartphone cameras, ensures that patients take their medications as prescribed, reducing dropout and missing data, improving the accuracy of clinical trial data and overall patient outcomes [140].

### 4.5. AI-Assisted Diagnosis and Computer Vision

Artificial intelligence has significantly improved the accuracy of Alzheimer’s disease diagnosis by automating the analysis of imaging data [141]. CNNs are routinely used to analyze CT and MRI scans, and detecting subtle structural changes in the brain, such as hippocampal atrophy, that are difficult to identify manually, becomes a very useful tool for radiologists [142]. For example, a study using DeepAD, a deep learning model, demonstrated its ability to detect hippocampal volume reduction with high accuracy, providing early markers of Alzheimer’s long before cognitive symptoms appear [143].

AI-powered image analysis enables the early identification of structural markers associated with Alzheimer’s, often years before symptoms manifest [144]. A notable example is VoxCNN, a CNN-based model that analyzes 3D brain images from MRI scans to identify Alzheimer’s-specific atrophy patterns. In clinical trials, VoxCNN has demonstrated superior performance in detecting early-stage Alzheimer’s compared to traditional diagnostic methods [145].

When comparing AI-assisted diagnoses to traditional methods, it is clear that AI provides more accurate, consistent, and reproducible results, reducing diagnostic delays [146]. In a clinical study, the DLAD (Deep Learning for Alzheimer’s Diagnosis) system outperformed radiologists in detecting early signs of Alzheimer’s from MRI scans, particularly in distinguishing between mild cognitive impairment and normal aging, which is often a challenge in traditional diagnostics because of the similarity of morphological differential diagnoses [147].

AI tools also support clinical decision making by providing comprehensive analytics that combine imaging data with genetic and clinical records [148]. For example, systems such as IBM Watson Health synthesize patient data from multiple sources, including genomic sequences, clinical history, and imaging results, to help diagnose Alzheimer’s and recommend the most appropriate treatments for each patient [149]. Another example is Neuroreader, an AI-powered software that analyzes MRI data to quantify brain atrophy and compare it to normative data, providing useful information for physicians during diagnosis [150].

In summary, Table 4 showcases the key areas where artificial intelligence has significantly impacted Alzheimer’s research. Each area of application utilizes specific AI techniques to achieve notable benefits while also facing certain challenges.

## 5. Conclusions

Recent studies on Alzheimer’s disease have shed light on new therapeutic targets and avenues for drug development. For decades, the amyloid hypothesis has been the cornerstone of drug development efforts, focusing on the accumulation of beta-amyloid in the brain. However, despite numerous clinical trials aimed at reducing amyloid burden, the results have been largely disappointing, raising questions about the role of amyloid as a primary cause of Alzheimer’s disease pathology. These results suggest that Alzheimer’s may be the result of multiple molecular dysfunctions rather than driven solely by amyloid accumulation.

Emerging evidence points to other critical factors, such as tau protein accumulation, neuroinflammation, mitochondrial dysfunction, and synaptic loss, as key contributors to the disease. Research has proposed that synaptic loss, rather than amyloid or tau accumulation, could be a pivotal factor in the early progression of Alzheimer’s disease. Furthermore, the discovery of new genes, such as TREM2, which is involved in microglial function and immune response, has introduced new targets for therapeutic intervention. Furthermore, the importance of mitochondrial dysfunction and other proteins, such as midkine and pleiotrophin, in the pathogenesis of Alzheimer’s disease is increasingly recognized. These findings underscore the need to explore alternative pathways in therapeutic development and to take a more holistic view of the underlying mechanisms of the disease.

AI has the potential to revolutionize Alzheimer’s drug development by enabling a faster and more efficient identification of drug targets and assisting in the design of clinical trials. AI has already dramatically accelerated the drug discovery process, particularly in identifying new targets and screening potential compounds. Advanced algorithms and deep learning models have improved the accuracy of virtual screening and molecular docking processes, helping to identify more effective therapies. However, challenges remain in applying AI to drug development, such as the need for high-quality, unbiased data and the interpretability of AI models.

Looking ahead, AI will play an increasingly important role in personalized medicine, particularly in tailoring treatments based on a patient’s genetic and clinical profile. For example, integrating AI with digital twin technologies has tremendous potential. Digital twins (virtual representations of patients) could simulate the progression of Alzheimer’s disease in real time, allowing researchers to test personalized treatments in a virtual environment before implementing them in clinical practice. This combination of AI and digital twins represents a promising frontier in the research and development of treatments for Alzheimer’s disease.

To fully realize the potential of AI, multidisciplinary collaboration between researchers in genetics, neuroscience, immunology, and AI will be essential. In addition, innovative clinical trial designs, personalized medicine approaches, and the development of new biomarkers for the early diagnosis and monitoring of disease progression are necessary. AI, by identifying complex patterns in the interactions between biological systems such as the gut microbiota, immune system, and neurons, could enable a deeper understanding of the onset and progression of Alzheimer’s disease.

In conclusion, the field of Alzheimer’s disease research is rapidly evolving, with emerging evidence on the underlying mechanisms of the disease and the potential for new therapeutic targets. Advances in AI and personalized medicine offer exciting opportunities for drug development and patient care. However, there are significant challenges, such as data heterogeneity, model interpretability, and the integration of multiple biological datasets, that need to be addressed to fully realize the potential of these technologies. Future research should focus on translational approaches to bring promising therapies to patients more quickly.

As research progresses, fundamental biological questions remain: to what extent can interactions between different systems, such as immune response, inflammation, and mitochondrial dysfunction, explain the onset and progression of Alzheimer’s disease? Could they be a viable alternative to AI?

## Figures and Tables

**Table 1:** Summary of Alzheimer’s disease hypotheses and therapeutic targets.

Hypothesis	Overview
Amyloid Hypothesis	**Key Mechanism:** Accumulation of β-amyloid peptides forming senile plaques, triggering neuroinflammation and synaptic dysfunction. **Evidence:** Identification of Aβ plaques; toxic soluble oligomers [20]. **Therapeutic Targets:** Enzymes regulating Aβ production and clearance; targeting oligomers.
Tau Hypothesis	**Key Mechanism:** Abnormal tau protein forms neurofibrillary tangles, disrupting microtubules and leading to neuronal death. **Evidence:** Presence of hyperphosphorylated tau; correlation with disease progression [64]. **Therapeutic Targets:** Inhibitors of tau aggregation; microtubule stabilizers.
Neuroinflammation Hypothesis	**Key Mechanism:** Chronic activation of microglia releases pro-inflammatory cytokines, damaging neurons. **Evidence:** Elevated cytokines and chemokines in AD brains; microglial activation [6]. **Therapeutic Targets:** Modulators of microglial activity; anti-inflammatory agents.
Mitochondrial Dysfunction Hypothesis	**Key Mechanism:** Impaired mitochondrial function leads to energy deficits and oxidative stress. **Evidence:** Mitochondrial abnormalities; increased oxidative damage in neurons [69]. **Therapeutic Targets:** Antioxidants; enhancers of mitochondrial biogenesis; mitophagy inducers.
Synaptic Dysfunction Hypothesis	**Key Mechanism:** Alterations in synaptic function impair neuron communication, leading to cognitive decline. **Evidence:** Synaptic loss observed in AD; links to cognitive deficits [49]. **Therapeutic Targets:** Agents restoring synaptic function; neurotrophic factors.
Oxidative Stress Hypothesis	**Key Mechanism:** Excess reactive oxygen species cause cellular damage and apoptosis. **Evidence:** Presence of oxidative damage in AD brains; lipid peroxidation [50]. **Therapeutic Targets:** Antioxidants; compounds reducing oxidative stress.
Neurovascular Hypothesis	**Key Mechanism:** Vascular dysfunction reduces cerebral blood flow, contributing to neurodegeneration. Evidence: Blood–brain barrier breakdown; reduced flow in AD patients [5]. **Therapeutic Targets:** Vascular health agents; improving blood–brain barrier integrity.
Endoplasmic Reticulum Stress Hypothesis	**Key Mechanism:** Accumulation of misfolded proteins induces ER stress and neuronal death. Evidence: Elevated ER stress markers; unfolded protein response activation [55]. **Therapeutic Targets:** Modulators of protein folding; ER stress reducers.
Epigenetic Hypothesis	**Key Mechanism:** Epigenetic modifications alter gene expression affecting neuronal function. Evidence: DNA methylation changes; histone modifications in AD brains [70]. **Therapeutic Targets:** Epigenetic modulators; drugs targeting gene expression.
Metal Hypotheses (Zinc and Aluminum)	**Key Mechanism:** Dysregulated metal ions contribute to protein aggregation and toxicity. Evidence: Altered metal levels in AD brains; metals interact with Aβ and tau [59]. **Therapeutic Targets:** Metal chelators; normalizing metal homeostasis.
Lifestyle Factors Hypothesis	**Key Mechanism:** Diet, physical activity, and sleep patterns influence AD risk. Evidence: Epidemiological links between lifestyle and AD incidence [61]. **Therapeutic Targets:** Lifestyle interventions; dietary modifications.

**Table 2:** AI training models.

AI Approach	Description
Supervised Learning	Supervised learning involves training models on labeled data to predict outcomes such as disease progression based on clinical data [90]. This method is useful for tasks such as prognosis estimation in Alzheimer’s patients.
Unsupervised Learning	Unsupervised learning is applied to analyze high-dimensional and unlabeled datasets, such as genomic and proteomic sequences [91]. It is particularly useful in discovering novel patterns, classifications, and subgrouping of patients without predefined labels.
Reinforcement Learning	Reinforcement learning is used to simulate and optimize therapeutic strategies by learning from the outcomes of previous simulations [92]. It is increasingly applied to find optimal treatment protocols and intervention strategies for neurodegenerative diseases.

**Table 3:** Description of AI models applied to Alzheimer’s disease.

Model	Description
SVM (Support Vector Machine)	A supervised learning algorithm that seeks an optimal hyperplane to separate data points of different classes with maximal margin [93]
RF (Random Forest)	An ensemble method composed of multiple decision trees. It reduces overfitting and increases predictive accuracy by averaging the results of numerous weak learners [93].
ANNs (Artificial Neural Networks)	Computational models inspired by biological neural networks, capable of recognizing complex patterns in data through interconnected layers of weighted nodes [94].
DNNs (Deep Neural Networks)	A type of neural network with multiple hidden layers that can automatically learn hierarchical representations, enabling the extraction of increasingly abstract features [95].
LBVS (Ligand-Based Virtual Screening)	A computational approach that uses known active compounds to predict new molecules with similar biological activity, facilitating the discovery of novel candidates [96].
Deep Learning Models	A family of advanced neural network architectures (including CNNs, RNNs, and transformers) that learn from large datasets, often without the need for manual feature engineering [97].
AlphaFold	An AI system that predicts protein structures from amino acid sequences with high accuracy, providing insights into molecular form and function [98].
Systems Biology Models	Integrative computational frameworks that combine data from various biological levels (genomes, proteomes, metabolomes) to model complex biological systems holistically [99].
Agent-Based Models	Simulation models where individual entities (agents) follow defined rules, allowing complex global patterns to emerge from local interactions [100].
Bayesian Networks	Probabilistic graphical models that represent variables and their conditional dependencies, enabling reasoning under uncertainty and probabilistic inference [101].
NLP (Natural Language Processing)	Techniques and models for analyzing, understanding, and generating human language, often applied to tasks like sentiment analysis, machine translation, and information extraction [102].
Sensor-Based Monitoring	The use of wearable devices, IoT sensors, and other technologies to continuously capture data streams (e.g., physiological signals, environmental parameters) for subsequent analysis [103].
DeepAD	A specialized deep learning approach designed for complex classification and prediction tasks, integrating multiple data types and learning representations at various scales [104].

**Table 4:** Summary of AI techniques in Alzheimer’s research.

Area of Application	AI Techniques Used	Key Benefits	Challenges
Virtual Screening of Active Compounds	SVM, RF, ANN, DNN, LBVS	Accelerated identification of potential drug candidates, improved screening efficiency	Data quality, computational resources [108]
Molecular Docking and Dynamics	Deep Learning Models (AlphaFold, DeepDock, AtomNet, CNNScore, RF-Score)	Enhanced accuracy in predicting protein structures and ligand interactions	Modeling biological flexibility, validation of predictions [113]
Modeling Disease Hypotheses	Systems Biology Models (EpiSim), Agent-Based Models (Repast Simphony), Bayesian Networks, Reinforcement Learning	Integrated understanding of disease mechanisms, identification of therapeutic targets	Complexity of biological systems, data integration [120]
Patient Monitoring in Clinical Studies	Wearable Devices, NLP, Sensor-Based Monitoring	Real-time data collection, personalized treatment plans, improved adherence	Data privacy, variability in patient data [134]
AI-Assisted Diagnosis	Deep Learning Models (DeepAD, VoxCNN, CNNs)	Early detection of structural brain changes, increased diagnostic accuracy	Requirement for large datasets, potential for overfitting [143]

## Abbreviations

The following abbreviations are used in this manuscript:

MDPI	Multidisciplinary Digital Publishing Institute
DOAJ	Directory of open access journals
TLA	Three-letter acronym
LD	Linear dichroism
AD	Alzheimer’s disease
Aβ	Amyloid-beta
ML	Machine learning
AI	Artificial intelligence
ROS	Reactive oxygen species
CNNs	Convolutional neural networks
LBVS	Ligand-based virtual screening
SVMs	Support vector machines
RFs	Random forests
ANNs	Artificial neural networks
DNNs	Deep neural networks
NLP	Natural language processing
